# The role of non-coding RNA regulates stem cell programmed death in disease therapy

**DOI:** 10.1016/j.ncrna.2025.04.005

**Published:** 2025-04-23

**Authors:** Ziling Liao, Weidong Liu, Lei Wang, Wen Xie, Chaoyan Yao, Qianping Huang, Xingjun Jiang, Caiping Ren

**Affiliations:** a Department of Neurosurgery, Xiangya Hospital, Central South University, China; b Xiangya School of Basic Medical Science, Central South University, China; c National Clinical Research Center for Geriatric Disorders, Xiangya Hospital, Central South University, Changsha, Hunan, 410008, China; d The Key Laboratory of Carcinogenesis and Cancer Invasion of the Chinese Ministry of Education, Cancer Research Institute, Xiangya School of Basic Medical Science, Central South University, Changsha, Hunan, 410078, China

**Keywords:** Programmed cell death, Non-coding RNA, Stem cells, Stem cell therapy, Disease

## Abstract

Programmed cell death (PCD), an essential and inevitable phenomenon, is integral to organismal development. It not only maintains cellular homeostasis but also prevents aberrant cell proliferation, thereby protecting normal growth and development from detrimental factors. And it is governed by a highly complex and sophisticated regulatory network in which non-coding RNAs (ncRNAs) play pivotal roles. ncRNAs refer to RNA molecules that do not encode proteins and encompass various types, including long non-coding RNA (lncRNA), microRNA (miRNA), and circular RNA (circRNA). Herein, we investigate the specific signaling mechanisms by which ncRNAs regulate stem cells, elucidating their role in modulating PCD process through interactions with specific molecules. We further summarize the impact of above modulating role on stem cell differentiation, proliferation, cycle regulation and diverse disease development and therapy. Additionally, given the emerging trends in the therapeutic application of ncRNAs and stem cells, we explore the potential of their combined application for disease treatment.

## 1 Introduction

PCD represents a kind of regulated cell death orchestrated by a series of molecular programs, playing a crucial role in the development and homeostasis of organisms. Initially, apoptosis was recognized as the sole form of PCD [1]. With the advancement of research, over ten distinct forms of PCD, including autophagy, proptosis, and necroptosis have been identified.

In the physiological process, stem cells undergo PCD to effectively eliminate damaged or senescent cells. This mechanism not only preserves the self-renewal capacity of stem cells but also ensures their potential for multi-lineage differentiation. During disease progression and treatment, various factors such as inflammatory mediators, environmental factors, and pharmacological effects may induce PCD-mediated clearance of stem cells [[2], [3], [4]]. Increasing evidence suggests that ncRNAs play a pivotal role in regulating PCD in stem cells and finely modulate stem cell fate.

ncRNAs are functional RNA molecules that generally do not encode proteins, representing more than 90 % of the RNA content in the human genome [5,6]. Prominent categories of ncRNAs include lncRNAs, miRNAs, and circRNAs. lncRNAs are distinguished by their length, which exceeds 200 nucleotides, and by their conserved secondary structures. Due to the lack or minimal presence of effective open reading frames, lncRNAs seldom, if ever, encode proteins [[7], [8], [9], [10], [11]]. circRNAs are single-stranded, covalently closed RNA molecules that are comparable in length to lncRNAs. They play various biological roles, including acting as transcriptional regulators, miRNA sponges, and protein templates [12]. miRNAs are short, single-stranded RNA molecules, typically ranging from 19 to 25 nucleotides in length, that regulate the post-transcriptional silencing of target genes. A single miRNA can target hundreds of mRNAs, thereby influencing gene expression within functional interaction pathways [13]. One of the pivotal mechanisms by which ncRNAs exert their regulatory roles is through intermolecular interactions. This involves the formation of specific complexes or conjugates between ncRNAs and other biomolecules, including proteins, DNA, and RNA, to orchestrate cellular activities. Functional interactions can also occur among ncRNAs themselves. For instance, lncRNAs or circRNAs can act as endogenous competitive RNAs (ceRNAs) for miRNAs by complementing and pairing with them, thereby hindering their binding to target mRNAs and ultimately regulating gene expression, chromatin structure, and cellular processes [14,15]. These regulatory mechanisms mediated by ncRNAs are crucial in the PCD of stem cells, either directly or indirectly, and are fundamentally significant for the origin, development, and maintenance of life.

In this review, we aim to comprehensively elucidate the pivotal roles of ncRNAs in stem cells PCD, along with the underlying intricate mechanisms of action. Through this endeavor, we aspire to attain a profound understanding of the significant involvement of ncRNAs in regulating stem cell PCD during disease onset and progression, thereby unveiling their potential applications in the field of stem cell therapy. These findings will offer novel insights and establish a foundation for technological advancements, stem cell therapy, and clinical applications.

## 2 ncRNAs modulate diverse forms of PCD in stem cells

### 2.1 Apoptosis

**Fig. 1:**
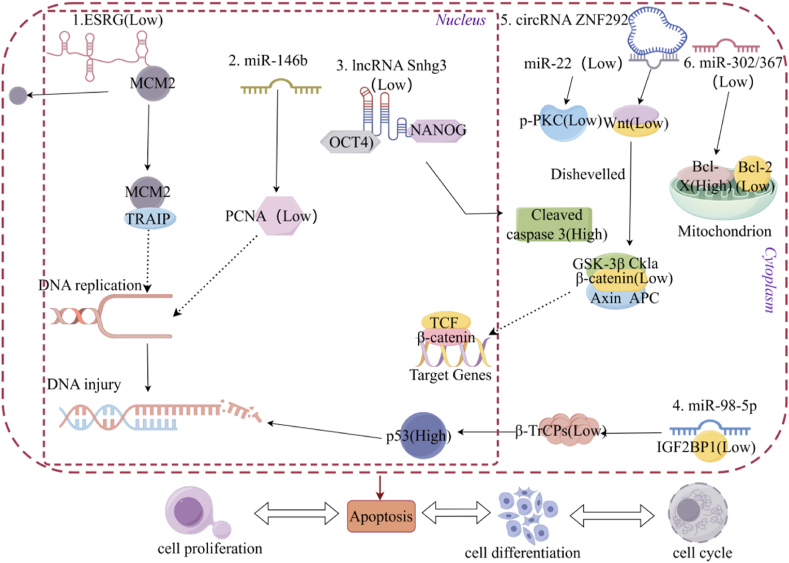
Part of the mechanisms of ncRNAs in regulating stem cell apoptosis.

Under physiological conditions, cell death predominantly occurs through apoptosis, a process that has been extensively investigated in the context of PCD in stem cells. Apoptosis can be initiated by both intrinsic and extrinsic pathways. The intrinsic pathway, also known as the mitochondrial apoptosis pathway, involves changes in mitochondrial membrane permeability in response to stimuli such as pharmacological agents or DNA damage. This process is stringently regulated by pro-apoptotic proteins belonging to the BCL-2 family [16]. The process of exogenous apoptosis is a regulated cellular demise, wherein the activation of caspase-8 is facilitated by Fas/FasL, tumor necrosis factor (TNFR1/TNFα), and its associated apoptosis-inducing receptor and ligand (TRAILR/TRAIL), as well as other death receptor pathways upon external stimulation. As depicted in Fig. 1 and Table 1, numerous studies have demonstrated the pivotal role of ncRNAs in regulating apoptosis of stem cell while being intricately linked to other cellular processes.

(1) In hESCs, the lncRNA ESRG interacts with the replication licensing factor-MCM2, thereby facilitating MCM2's nuclear localization and protein stability. Upon reduction in the level of ESRG, MCM2 undergoes ubiquitination and subsequent degradation, and its partial translocation from the nucleus to the cytoplasm. Consequently, DNA replication is disrupted, resulting in cellular DNA damage;
(2) In bovine male germline stem cells, the expression of PCNA mRNA is inhibited by miR-146b, leading to impaired DNA synthesis and concurrent occurrence of apoptosis while cell proliferation is suppressed;
(3) In mESCs, lncRNA Snhg3 interacts with pluripotent markers OCT4 and NANOG. Suppression of Snhg3 induces cellular differentiation, accompanied by the activation of caspase 3 to facilitate apoptosis;
(4) In MSCs, miR-98-5p downregulates the expression of insulin-like growth factor 2 mRNA binding protein 1 (IGF2BP1), resulting in the degradation of ubiquitin ligase-β-transducin repeat-containing protein (β-TrCPs) and subsequent activation of the p53 signaling pathway by inhibiting p53 protein ubiquitination;
(5) In NSCs, the circRNA ZNF292 functions as a ceRNA for miR-22 and exerts its regulatory effects by down-regulating the protein levels of Wnt and Phosphorylated Protein Kinase C (p-PKC). Consequently, it simultaneously modulates the signaling pathways of Wnt/β-catenin and PKC/Extracellular Signal-Regulated Kinase (ERK);
(6) After the decrease of miR-302/367, there is a down-regulation in the expression of the anti-apoptotic protein BCL-xL, and an up-regulation in the level of the pro-apoptotic protein BNIP3L.

**Table 1:** The pertinent targets and mechanisms through which ncRNAs regulate apoptosis.

ncRNAs	Role in apoptosis	Mechanism of action	Reference
lncRNA CASC7	Inhibits the early apoptosis of cells	Competes with miRNA-122-5p to inhibit the action of the transcription factor CBL	[18]
ESRG	Inhibits DNA injury and the early apoptosis of cells	Combined with MCM2, HNRNPA1	[19]
lnc530	Reduces the formation of R-loops during transcription, thus reducing DNA damage	Forms complexes with DDX5 and TDP43 to enhance R-loop homeostasis regulation	[20]
miR-302/367	Inhibits apoptosis	Down-regulates BNIP3L/Nix and up-regulating the expression of BCL-xL	[22]
Bta-miR-146b	Promotes cell apoptosis while inhibiting proliferation	Reduces PCNA, cyclinD1, p21 and CDK2 mRNA levels, while increase Bax and Bcl-2 protein levels	[23]
	Leads to apoptosis of NSCs	Inhibits of PROK2 function by PBX1	[28]
lncAABR07053481	Inhibits hypoxia-induced apoptosis	Inhibits miR-664-2-5p, thereby alleviating the silence-inhibiting effect of miR-664-2-5p on Notch1, promoting the expression of anti-apoptotic proteins such as Bcl-2	[30]
miR-21, miR-23 and miR-210	Promotes the survival of MSCs under hypoxic condition and prevent cell apoptosis		[32]
miR-10a	Reduces hypoxia-induced apoptosis of senescent hBM-MSCs	Inhibits KLF4-bax/BCL2 pathway	[33]
miR-210-3p	Inhibits apoptosis	Targets CDIP1 and reduces its expression	[34]
miR-181a-5p	Promotes apoptosis	Regulates of Sirt1 and affects PI3K/Akt signaling pathway	[38]
lncRNA RAD51-AS1	Inhibits apoptosis	Interacts with YBX1 and activates TGF-β signaling by binding Smad7 and Smurf2 mRNA	[39]
lnc IGF2AS	Promotes apoptosis		[40]
lncRNA NORAD	Promotes dexamethasone-induced apoptosis	Inhibits the expression of miR-26a-5p	[41]
miR-145	Promotes dexamethasone-induced apoptosis		[42]
linc00324	Promotes apoptosis	Regulates the expression of the miR-7977, STK4 and downstream signaling pathways	[43]
lnc GAS5	Inhibits apoptosis		[44]
circRNA_014511	Inhibits apoptosis	Binds to miR-29b-2-5p	[45]
circRNA_014511	Promotes irradiation-induced apoptosis	Regulates the miR-1249-5p/HIPK2 axis	[46]

#### 2.1.1 ncRNAs regulate apoptosis of stem cells by influencing the process of DNA synthesis, replication and metabolism

The development of many diseases involves changes in the DNA level. As previously discussed, DNA damage constitutes a primary cause of endogenous apoptosis [17]. ncRNAs play a regulatory role in critical processes such as DNA synthesis, replication, and subsequent processing and modification. For example, the expression level of lncRNA CASC7 in sperm-derived stem cells from patients with obstructive azoospermia (OA) is comparatively lower than that observed in healthy individuals. As a molecular sponge for miRNA-122-5p, it facilitates the inhibition of cell proliferation and DNA synthesis by the downstream protein CBL (Cbl proto-oncogene), thereby impeding cell division and DNA synthesis while promoting early apoptosis [18]. During the DNA replication phase, ncRNAs have the capacity to bind to and regulate factors involved in replication licensing, thereby influencing the response to and processing of DNA damage. In our previous investigation, we identified that the lncRNA ESRG (embryonic stem cell related gene), which is abundantly expressed in human embryonic stem cells (hESCs), interacts with minichromosome maintenance complex component 2 (MCM2). This interaction is crucial for maintaining steady-state levels and nuclear localization of MCM2, thereby ensuring accurate DNA replication. The suppression of ESRG expression resulted in the ubiquitination and proteasome-mediated degradation of MCM2, along with the up-regulation of proteins such as Cytochrome C, BAX, Cleaved-caspase 3, and Cleaved-caspase 7. Additionally, the suppression of ESRG expression caused the translocation of MCM2 from the nucleus to the cytoplasm and enhanced the interaction between E3 ubiquitin ligase-TRAF Interacting Protein and MCM2. Consequently, this promoted the ubiquitination of MCM2, leading to the activation of the cellular DNA damage response and triggering apoptosis [19]. In addition to their roles in DNA synthesis and replication, ncRNAs have the capacity to modulate stem cell apoptosis by influencing DNA metabolic pathways. R-loops, which are defined by their dynamic triple-stranded nucleic acid structure, serve as critical intermediates in DNA metabolism and can induce replication stress, thereby contributing to DNA damage. Lnc530 is situated within the R-loop and interacts with DEAD-box helicase 5 (DDX5) and TAR DNA-binding protein (TDP43), thereby augmenting local protein concentrations and enhancing the steady-state regulation of the R-loop while concurrently diminishing R-loop formation during transcription. Deletion of lnc530 leads to the accumulation of R-loops and subsequent DNA damage in MSCs [20]. These studies have elucidated the multifaceted mechanisms by which ncRNAs regulate DNA damage and influence apoptosis of stem cell. Within these mechanisms, various signaling pathways are either activated or inhibited.

#### 2.1.2 ncRNAs affect all of stem cell apoptosis, differentiation, cycle and proliferation

The normal operation of biological processes such as cell differentiation, cycle and proliferation is one of the basic guarantee conditions for stem cell therapy.

And the modulation of stem cell apoptosis by ncRNAs constitutes a complex and interconnected process with implications that extend beyond apoptosis itself, encompassing various other biological processes such as cell differentiation. For example, the ESRG mentioned above not only regulates apoptosis and differentiation through MCM2 but also interacts with and stabilizes the heterogeneous ribonucleoprotein A1 (HNRNPA1) protein, thereby modulating the alternative splicing of TCF3, influencing CDH1 expression, and maintaining the self-renewal and pluripotency of hESCs. Decreased the expression of ESRG leads to cellular differentiation phenotype, reduced alkaline phosphatase (AP) activity, and downregulation of pluripotency marker genes such as SOX2 and OCT4 [21]. Therefore, when using the differentiation ability of stem cells for stem cell therapy, the effect of apoptosis also needs to be considered.

In addition to its influence on cellular differentiation, the regulatory function of ncRNAs in stem cell apoptosis is intricately associated with cell cycle progression. The miR-302/367 cluster exhibits dose-dependent dual regulation of the cell cycle and apoptosis in hESCs. A reduction of 40 % in the expression level of this cluster is sufficient to induce G0/G1 phase arrest and decrease the proportion of S-phase cells. Further reduction to 20 % results in a marked decline in both G0/G1 and S-phase populations, an increase in G2/M-phase cells, and a significant elevation in the rate of apoptosis. Mechanistically, the miR-302/367 cluster downregulates BNIP3L (a BH3-only proapoptotic factor)/Nix, and upregulates BCL-xL expression to counteract apoptosis [22]. Therefore, the normal operation of ncRNAs regulation cycle is closely related to the occurrence of apoptosis.

Apoptosis and cell proliferation are fundamental biological processes that collectively regulate the dynamic equilibrium of cellular populations *in vivo*. ncRNAs play a pivotal role in maintaining these balances. For instance, in germline stem cells, the overexpression of miR-146b results in a decrease in the mRNA levels of proliferating cell nuclear antigen (PCNA), while concurrently increasing the protein levels of Caspase-9, and decreasing the expression ratio of Bcl-2/Bax [23]. This molecular alteration promotes apoptosis and inhibits proliferation, thereby supporting the hypothesis that apoptosis functions as a mechanism to maintain cellular homeostasis by balancing cell proliferation.

In conclusion, ncRNAs have the capacity to dynamically modulate cellular fate by affecting the activity, stability, or interactions of key proteins. This regulation ensures that organisms maintain appropriate cell numbers and function during development and tissue homeostasis. It is beneficial to the further exploration of stem cell therapy.

#### 2.1.3 The role of ncRNA in regulating apoptosis in hypoxia and glucose deprivation

*In vitro* studies often utilize hypoxia and glucose deprivation to simulate brain injury, inflammation, and various disease models [[24], [25], [26], [27]]. Under conditions of glucose deprivation and hypoxia, the role of ncRNAs in apoptosis is multifaceted. Certain genes facilitate apoptosis during disease progression. For example, apoptosis induced by miR-141-3p in neural stem cells can be counteracted through the overexpression of its downstream protein, PBX homeobox 1 (PBX1). Further studies have demonstrated that PBX1 mediates this counteractive effect by inhibiting the function of PROK2 (prokineticin 2), an insulin-induced risk factor for cerebral ischemic injury. These findings provide novel insights and strategies for the management of middle cerebral artery occlusion [28]. And ncRNAs also play a crucial role in cellular resistance to adverse conditions and the inhibition of apoptosis. For instance, the upregulation of LncTmem235 competes with miR-34a-3p for binding to the mRNA of baculoviral IAP repeat containing 5 (BIRC5), an apoptosis-inhibiting gene. This competitive binding enhances the expression of BIRC5, thereby inhibiting hypoxia-induced apoptosis in BMSCs. When BMSCs transplantation was utilized for *in vitro* treatment of osteonecrosis, transfection of LncTmem235 into the cells significantly facilitated the repair of defect areas [29]. In addition, LncAABR07053481 [30], miR-222 [31], miR-21, miR-23a, miR-210 and miR-10a [32,33] can also promote the survival of MSCs under hypoxic conditions and protect cells from apoptosis. Nevertheless, some studies have demonstrated that hypoxic preconditioning can enhance the survival and therapeutic potential of stem cells. For example, transient induction of hypoxia results in a dose-dependent elevation in the expression levels of miR-210-3p. Further studies have demonstrated that miR-210-3p targets the key mediator of apoptosis, and inhibiting the Bid/Bax/cleaved caspase3-mediated extrinsic apoptotic pathway cell survival and functional stability are promoted under angiotensin II conditions [34]. It can be seen that to optimize the therapeutic effect, by regulating the effect of ncRNAs on the stem cell apoptosis process, we can carefully select and apply appropriate external conditions to maximize the regulatory role of ncRNAs, to achieve better therapeutic effect.

The regulation of apoptosis by ncRNAs under oxygen-glucose deprivation/reperfusion (OGD/R) conditions involves a series of complex genes and signaling pathways, characterized by intricate interaction mechanisms and extensive regulatory networks. The p53 signaling pathway is one of the important regulated members of ncRNAs. The inhibition of H19 in endogenous neural stem/progenitor cells (NSPCs) can enhance the expression of p53, thereby promoting apoptosis. Conversely, the effects of H19 overexpression can be mitigated by the upregulation of p53, suggesting an antagonistic interaction between H19 and the p53 signaling pathway in the regulation of apoptosis, abolished OGD/R-induced NSPCs apoptosis [35]. In addition, ncRNAs are capable of simultaneously modulating multiple signaling pathways, thereby influencing stem cell apoptosis. For instance, circular RNA cZNF292 exerts a negative regulatory effect on miR-22 expression and activates both the Wnt/β-catenin and PKC/ERK signaling pathways. Inhibition of miR-22 expression results in a concomitant decrease in Wnt3a and β-catenin levels, as well as a reduction in the phosphorylated PKC/ERK ratio, which collectively promote apoptosis in NSCs under conditions of oxygen-glucose deprivation. And it furnished the theoretical basis for further research on Hypoxic-ischaemic encephalopathy progression [36]. Under the same conditions, the regulatory mechanism by which H19 influences apoptosis involves the activation of both the PI3K/AKT and Wnt/β-catenin pathways [37]. These results indicate that when using ncRNAs combined with stem cells to treat diseases under glucose and oxygen deprivation conditions, multiple signaling pathways can be focused at the same time to achieve a more efficient cure effect.

#### 2.1.4 The role of ncRNA in regulating apoptosis in osteoporosis

In addition to the above results, ncRNAs can also regulate other signaling pathways involved in the treatment of osteoporosis.Notably, the PI3K/Akt signaling pathway is of considerable significance. For example, miR-181a-5p modulates the PI3K/Akt signaling pathway by targeting Sirtuin 1 (Sirt1). Elevated expression levels of Sirt1 in MSCs result in an increased ratio of p-PI3K/PI3K and p-Akt/Akt, however, concurrent overexpression of miR-181a-5p produces opposing effects, accompanied by a heightened rate of apoptosis. This process provides a new target for osteoporosis treatment [38]. Additionally, ncRNAs play a crucial role in modulating the TGF-β signaling pathway, thereby significantly impacting stem cell apoptosis. For instance, the lncRNA RAD51-AS1 in hBMSCs from patients with osteoporosis was significantly lower than those from healthy donors, it can interact functionally with Y-box binding protein 1 (YBX1), which binds to Smurf2 mRNA. Smurf2 not only associates with Smad7 to form an E3 ubiquitin ligase complex that targets components of the TGF-β signaling pathway for degradation but also negatively regulates the ubiquitination within the TGF-β signaling pathway. Therefore, the inhibition of lncRNA RAD51-AS1 results in the activation of the TGF-β signaling pathway, consequently elevating the apoptosis rate of hBMSCs [39]. In summary, the complexity of ncRNAs in regulating apoptosis is demonstrated through their interference with diverse signaling pathways such as PI3K/Akt, Wnt/β-catenin and TGF-β, and these signaling pathways influence and coordinate with each other. In summary, the treatment of osteoporosis involves a variety of genes, and the complexity of ncRNAs regulating apoptosis is reflected in their interference with multiple signaling pathways such as Wnt/β-catenin and TGF-β, which may interact and interact with each other. In the treatment of osteoporosis, the use of ncRNAs to regulate the above pathways to reduce the apoptosis rate of stem cells provides an alternative treatment option.

#### 2.1.5 The role of ncRNA in regulating apoptosis in therapeutic strategies

The regulatory function of ncRNAs in stem cell apoptosis has been implicated in various therapeutic strategies for disease. For example, the inhibition of IGF2AS can activate the BDNF/Akt signaling pathway, thereby protecting NSCs from anesthesia-induced neurotoxicity [40]. Moreover, both lncRNA NORAD and miR-145 have been shown to enhance dexamethasone-induced apoptosis of BMSCs, offering a potential pharmacological approach for targeted ncRNA inhibition in the treatment of steroid-induced femoral head necrosis [41,42]. In addition to its involvement in the pharmacological effects of drugs, ncRNAs plays a critical regulatory role in the utilization of specific materials and methods for the treatment of certain diseases. For example, linc00324 is implicated in the miR-7977/STK4 pathway-mediated anti-apoptotic effect of graphene oxide on Ad-MSCs. The combination of graphene oxide and linc00324 knockdown exhibits enhanced auxiliary properties for promoting diabetic wound healing [43]. The application of a heparin sulfate-collagen scaffold in the treatment of spinal cord injury, combined the transfection of the lncRNA-GAS5 plasmid with 3D-printed iPS-NSCs scaffold, significantly enhances the activity of iPS-induced NSCs by the fifth day. This intervention attenuates neuronal apoptosis and injury while promoting regenerative capacity in spinal cord injury. In contrast, silencing GAS5 negates these beneficial effects [44]. Whole body irradiation combined with chemotherapy pretreatment is commonly employed prior to hematopoietic stem cell transplantation. In this process, circRNA-014511 can be utilized to mitigate the apoptosis rate following radiation and enhance radiotherapy resistance by binding to miR-29b-2-5p, suppressing p53 expression, and promoting BMSCs survival [45]. Conversely, Circ-016901 facilitates irradiation-induced apoptosis [46]. To summarize, the synergistic integration of ncRNAs with other therapeutic strategies holds great potential in enhancing treatment efficacy and provides a novel perspective for exploring innovative and efficient therapeutic options.

### 2.2 Autophagy

**Fig. 2:**
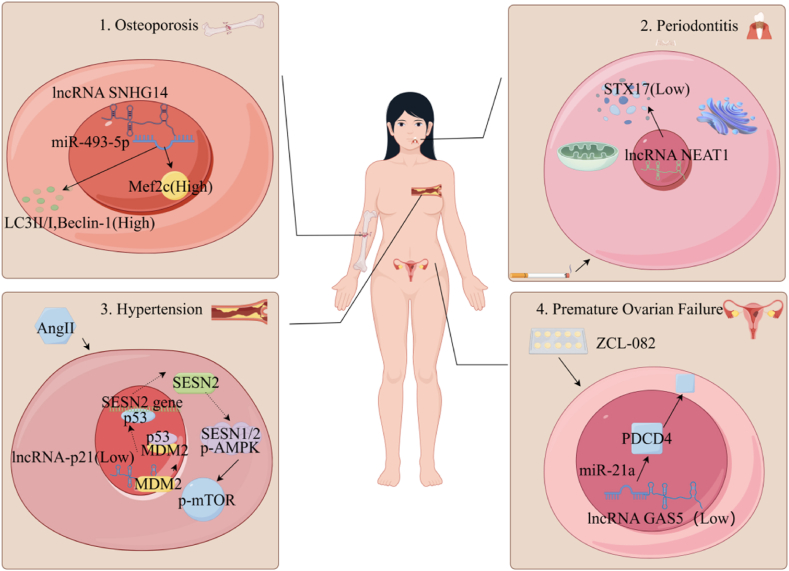
Part of the mechanism by which ncRNAs regulate autophagy is related to disease.

Autophagy is an intrinsic catabolic process that facilitates the regulated degradation of superfluous or impaired cellular constituents. Additionally, it functions as a vital defense mechanism that allows cells to endure various stress conditions, such as nutrient or growth factor deprivation, hypoxia, reactive oxygen species (ROS), DNA damage, and intracellular pathogens [47]. Upon exposure to the previously mentioned stress conditions, cellular responses involve the activation of AMPK and mTOR (mammalian target of rapamycin) signaling pathways, which subsequently initiate autophagy. Recent research on autophagy has primarily focused on its implications in disease pathogenesis, particularly the regulatory role of ncRNAs in the mechanism of autophagy. This association between ncRNAs and autophagy not only sheds light on the development and progression of diseases but also presents novel perspectives and potential therapeutic targets for disease management (Table 2) (Fig. 2).

(1) In the context of osteoporosis treatment, the activation of lncRNA SNHG14 results in elevated levels of Myocyte Enhancer Factor 2C (Mef2c) protein, as well as an increased expression ratio of LC3 II/I and Beclin-1 within BMSCs. This process induces autophagy, thereby mitigating the symptoms of the disease;
(2) The mist produced by smoke combustion is characterized by a high concentration of nicotine, which has the potential to induce autophagy and contribute to the pathogenesis of periodontitis. Nonetheless, the overexpression of lncRNA NEAT1 has been demonstrated to effectively reduce STX17 (Syntaxin 17) protein levels, thereby inhibiting PDLSCs autophagic processes;
(3) Upon stimulation with AngII in EPCs, the expression of lncRNA-p21 was downregulated, which subsequently enhanced MDM2-mediated inhibition of the p53 protein. As a result, the expression of the Sestrin 2 (SESN2) gene was reduced, leading to a diminished formation of the SESN1/2, AMPK, and Tuberous Sclerosis Complex 2 (TSC2) complex. This reduction further suppressed mTOR phosphorylation and autophagy;
(4) The administration of ZCL-082, an anti-infective agent, has been observed to induce FGSCs autophagy, subsequently leading to premature ovarian failure in women. This phenomenon is mediated through the down-regulation of the lncRNA GAS5. GAS5 functions as a competing endogenous RNA for miR-21a, thereby indirectly augmenting the intracellular expression levels of programmed cell death protein 4 (PDCD4).

**Table 2:** Targets and mechanisms of ncRNAs regulating autophagy.

ncRNAs	Role in autophagy	Mechanism of action	Reference
lncRNA SNHG14	Activates autophagy	Regulates Mef2c through miR-493-5p	[49]
miR-152-5p	Inhibits autophagy	Targets Atg14 to reducing endogenous ROS accumulation and maintaining cell REDOX homeostasis	[50]
lncRNA NEAT1	Reduces autophagy in periodontal stem cells	Down-regulates of STX17 after nicotine treatment	[51]
H19	Enhances the anti-inflammatory ability of MSCs and inhibit autophagy	Sponges adsorption of miR-138-5p and miR-141-3p	[53]
circCDK8	Induces autophagy and apoptosis of PDLSCs	Regulates the mTOR signaling pathway	[52]
miRNA 7k	Activates macrophage/autophagy	Promotes the expression of BECN1	[54]
lncRNA-p21	Enhances autophagy of EPCs	Promoting the transcriptional activity of p53 to activates SESN2/AMPK/TSC2 pathway	[55]
lncRNA WTAPP1	Inhibits autophagy	Sponges adsorption of miR-3120-5p, regulates MMP-1 expression via the PI3K/Akt and autophagy pathways	[57]
lncRNA Obox4-ps35	Decreases the rate of apoptosis and autophagy		[60]
miR-452-3p	Inhibits autophagy	Reduces Atg14 protein levels	[61]
lnc GAS5	Inhibites of autophagy of female germline stem cells (FGSCs)	As a ceRNA for miR-21a and reduces the level of PDCD4 protein	[65]
miR-326	Promotes autophagy of olfactory mucosal MSC and delayes cell senescence	Inhibits PTBP1 through PI3K signaling pathway	[66]
miR-148a		Regulates miR-148a/DNMT1/OCT4 autophagy pathway	[67]
circ-ZNF236	Promotes autophagy and the osteogenic differentiation of SCAPs.	Targets miR-218-5p and increases LGR4 expression	[69]
lncRNA FER1L4	Increase the formation of autophagosome and autolysosomes in PDLSCs	Inhibits of AKT phosphorylation	[71]
miR-34a	Inhibits autophagy	Combines with Syt1 and Atg9a	[72]

#### 2.2.1 The role of ncRNA regulating autophagy of stem cells in the pathogenesis and treatment of osteoporosis

Osteoporosis is a progressive skeletal disorder characterized by the impaired functionality of BMSCs. As the disease advances, the differentiation capacity of osteoblasts is hindered, resulting in a reduced osteoblast count, diminished activity, and a decreased rate of bone formation. Proper autophagic processes play a crucial role in mitigating the onset of osteoporosis. Jingbo Xue et al. found that the level of lncRNA SNHG14 increased in a time-dependent manner during the pathogenesis of osteoporosis, and regulated the expression of Mef2c through miR-493-5p, while P38/Mef2c was proved to be associated with autophagy activation [48]. The activation of SNHG14 or the inhibition of miR-493-5p expression leads to an upregulation of Mef2c protein levels, along with an increased LC3II/I and Beclin-1 expression ratio. Consequently, this induces autophagy and mitigates the reduction in bone mineral density and the Bone mass/total volume (BV/TV) ratio, thereby alleviating disease symptoms [49]. Autophagy-related proteins are also significant targets of ncRNAs. For example, Atg (Autophagy related genes)14 is directly targeted by miR-152-5p. The downregulation of miR-152-5p can enhance the expression of key autophagy proteins such as ALP, RUNX2, and OCN, reduce endogenous ROS accumulation, and maintain cellular redox homeostasis, ultimately regulating osteogenic differentiation [50]. These studies elucidate that ncRNA-mediated regulation of autophagy is intricately linked with bone differentiation, thereby providing novel therapeutic insights and potential interventions for osteoporosis.

#### 2.2.2 The role of ncRNA regulating autophagy of stem cells in the pathogenesis and treatment of inflammation

Autophagy is intricately associated with stem cell-mediated inflammation, and ncRNAs present promising therapeutic targets in this context. For example, lncRNA NEAT1 has been shown to downregulate STX17, thereby attenuating autophagy in periodontal ligament stem cells (PDLSCs). This mechanism is crucial to produce inflammatory factors following nicotine exposure from smoking [51]. Similarly, circCDK8 has been found to induce autophagy in PDLSCs via the mTOR signaling pathway under periodontitis conditions [52]. The H19 gene also plays a crucial role in regulating autophagy induced by inflammation via the PI3K/AKT/mTOR signaling pathway. Overexpression of H19 in MSCs led to elevated protein levels of focal adhesion kinase (FAK), 3-phosphoinositide-dependent protein kinase-1 (PDK1), p-AKT, p-mTOR, and sequestosome 1 (P62). Additionally, H19 upregulated the expression of protein tyrosine kinase 2 (PTK2, which encodes FAK), thereby enhancing the anti-inflammatory capacity of MSCs and inhibiting autophagy by sequestering miR-138-5p and miR-141-3p through sponge mechanisms [53]. Furthermore, within the framework of colitis treatment via stem cell transplantation, miRNA 7k inhibits the STAT3 signaling pathway in MSCs. STAT3 specifically interacts with the beclin 1 gene, recruiting histone deacetylase 3 (HDAC3) to repress BECN1 transcription. As a result, miRNA 7k indirectly promotes BECN1 expression, thereby inducing autophagy [54]. Therefore, attenuating the function of miRNA 7k may enhance therapeutic efficacy under stressful conditions. The findings of these studies indicate that distinct approaches are necessary to achieve anti-inflammatory effects when treating various inflammations with ncRNAs and stem cells.

#### 2.2.3 The role of ncRNA regulating autophagy of stem cells in the pathogenesis and treatment of hypertension and thrombosis

Autophagy is also critically involved in the therapeutic management of numerous diseases. Notably, the application of endothelial progenitor cells (EPCs) for the treatment of vascular injury has become a significant area of investigation in the context of stem cell therapy for hypertension. The impairment of EPCs is a crucial concern in hypertensive patients, and one important mechanism for protecting EPCs from damage involves reversing the declining trend of autophagy. In response to AngII, which is a primary factor contributing to vascular remodeling in hypertension, there is a noted reduction in the expression of lncRNA-p21 within EPCs. However, augmenting the expression levels of lncRNA-p21 can effectively bind to MDM2, thereby mitigating its inhibitory effect on p53. This interaction subsequently enhances the transcriptional activity of p53, leading to the induction of SESN2 expression and ultimately promoting the activation of AMP-activated protein kinase (AMPK). Consequently, SESN1/2 interacts with phosphorylated AMPK and TSC2 to form a complex, resulting in the inhibition of mTOR phosphorylation and the promotion of EPCs autophagy. This mechanism is crucial for safeguarding EPCs against AngII-induced cellular damage, thereby playing a significant role in the treatment of hypertension [55]. However, the role of lncRNA TUG1 is to promote autophagy and restore the function of EPCs treated with high glucose [56]. The process of vascular differentiation of EPCs is also an important event in thrombosis, and the autophagy pathway is actively involved. For instance, lncRNA WTAPP1 regulates the expression of miR-3120-5p, thereby modulating the level of matrix metallopeptidase 1 (MMP-1) protein and regulating angiogenesis by modulating the PI3K/Akt/mTOR autophagy signaling pathway [57]. Similarly, miR-205 also regulates the aforementioned signaling pathways to promote angiogenesis and mitigate deep vein thrombosis [58]. It can be deduced that ncRNAs exert a substantial influence on the functionality of EPCs through the regulation of autophagy, a process that is crucial in the therapeutic strategies for angiogenesis and thrombosis.

#### 2.2.4 ncRNAs are involved in drug-induced stem cell autophagy

ncRNAs also have the capability to modulate drug-induced autophagy in stem cells. For example, all-trans retinoic acid (ATRA) has been recognized as a potential therapeutic agent for liver disease, specifically targeting liver progenitor cells. Furthermore, the miR-200a-3p molecule has been shown to counteract the autophagic response elicited by ATRA treatment [59]. Triptolide demonstrates significant anti-tumor and anti-inflammatory properties. Its administration leads to a marked upregulation of lncRNA Obox4-ps35 in the mouse sperm cell line GC2. Conversely, suppression of lncRNA Obox4-ps35 expression in GC2 cells results in decreased cell survival rates and elevated levels of apoptosis and autophagy, thereby intensifying the detrimental effects induced by triptolide. This study offers preliminary evidence supporting the reproductive toxicity associated with triptolide [60]; Moreover, CHNQD-00603, an alkaloid derived from marine organisms, demonstrates the capacity to induce autophagy. However, the upregulation of miR-452-3p in BMSCs leads to a significant 0.1-fold reduction in ATG14 expression, which markedly diminishes the formation of autophagosomes and consequently attenuates the biological effects mediated by CHNQD-00603 [61]. These findings indicate that ncRNAs may serve as promising tools for modulating drug side effects and effectively alleviating patient discomfort during therapeutic interventions.

#### 2.2.5 The role of ncRNAs regulate autophagy of stem cells in aging-related diseases

Autophagy maintains cellular homeostasis and repair capacity by degrading damaged cellular structures and recycling degradation products, thereby counteracting cellular senescence. The decline in autophagic activity during cell senescence exacerbates cellular damage and dysfunction [62]. Cellular senescence is a fundamental mechanism implicated in the pathogenesis of various diseases. Modulating the autophagy pathway presents a promising therapeutic strategy to mitigate senescence and address related disorders. Compared to youthful MSCs, senescent cells demonstrate a substantial reduction in autophagic activity and a pronounced increase in miR-142 expression levels. This result directly inhibits the expression of endothelial PAS domain protein 1 (Epas1), a regulator of autophagy, resulting in dysregulated autophagy and the accumulation of peroxisomes. These findings suggest a potential therapeutic target for age-related diseases associated with stem cell degeneration [63]. Proper autophagic activity is essential for the normal functioning of stem cells. Pharmacological restoration of autophagy in stem cells can partially sustain cellular vitality, thereby offering novel prospects for research and treatment related to aging [64]. ZCL-082, a benzoborazole derivative, exhibits promising potential as an anti-infective agent by inducing autophagy in female germline stem cell lines (FGSCs). Bo Li et al. discovered that ZCL-082 plays a role by down-regulating lncRNA GAS5. GAS5 functions as a ceRNA for miR-21a and its high expression level increased PDCD4 levels in cells, thereby rescuing FGSCs autophagy induced by ZCL-082. This mechanism could serve as a therapeutic target for treating premature ovarian failure and represents one of the pathways through which ZCL-082 impacts ovarian function [65]. The aging process following stem cell transplantation poses a significant limitation in the treatment of cerebral hemorrhage. miR-326 regulates autophagy by modulating the PI3K signaling pathway and polypyrimidine tract-binding protein 1 (PTBP1). Through its influence, PTBP1 and p-PI3K/PI3K protein levels are markedly reduced, thereby promoting autophagy of transplanted olfactory mucosa-derived mesenchymal stem cells (OM-MSCs). It is one of the reasons that hypoxic preconditioning improves cell senescence [66]. These present study elucidates the complex dualistic role of ncRNAs in regulating stem cell autophagy-induced aging, with implications for disease intervention. Various ncRNAs exert regulatory roles in either promoting or inhibiting stem cell senescence, while appropriate autophagy can sustain or enhance stem cell activity; however, excessive autophagy signifies the process of aging. The central challenge lies in effectively harnessing this dual mechanism to develop innovative therapeutic strategies, thereby addressing a critical issue in contemporary disease management.

#### 2.2.6 The relationship between the mechanism of ncRNAs regulating autophagy in stem cells and development

The occurrence of autophagy is intricately linked to cellular and sexual development, exemplified by the distinct regulatory pathways for miR-148a/DNMT1/OCT4 autophagy in Wharton jelly multipotent stem cells (WJ-MSCs) of different genders [67]. The involvement of ncRNAs in the regulation of autophagy also exerts a significant impact on tooth formation and remodeling. For example, The induction of odontogenic differentiation in stem cells from human exfoliated deciduous teeth (SHED) and stem cells from the apical papilla (SCAPs) can be achieved by IGFBP7-AS1 and circ-ZNF236, respectively, through the process of autophagy [68,69]. Orthodontic treatment utilizes mechanical force to induce tooth movement, which is associated with the remodeling and regeneration of periodontal tissues. In response to mechanical force, lncRNA FER1L4 in PDLSCs exhibits a significant upregulation, emerging as one of the most prominently upregulated transcripts [70], concurrently, the number of autophagosomes and autolysosomes in the cells also increases. The elevated expression of FER1L4 inhibits the phosphorylation of AKT. Notably, the AKT pathway serves as a negative regulator of autophagy. FER1L4 prevents mTOR activation by AKT and consequently suppressing mTOR pathway activity. These intricate series of biological molecular events collectively orchestrate the initiation of autophagy cascades [71]. In addition to regulating tooth formation and remodeling, The regulatory role of ncRNAs in autophagy also exerts a significant impact on neuronal differentiation. MiR-34a exhibits a negative impact on the process of neuronal differentiation. Synaptotagmin 1 (Syt1) and ATG9a are markedly upregulated during neuronal differentiation, both being targets of miR-34a. Notably, ATG9a is essential for supplying membranes necessary for autophagosome formation. The regulatory impact of miR-34a on these molecules is critical for autophagy and neurogenesis. Subsequent studies have demonstrated that the expression of ATG9a is increased upon the induction of autophagy by rapamycin, consequently augmenting the number of Atg9 vesicles and partially mitigating the adverse effects imposed by miR-34a on neuronal differentiation [72]. The effect of stem cell autophagy regulated by ncRNAs on differentiation is not only reflected in local development, but also in the overall development of individual gender.

### 2.3 Other forms of PCD

**Fig. 3:**
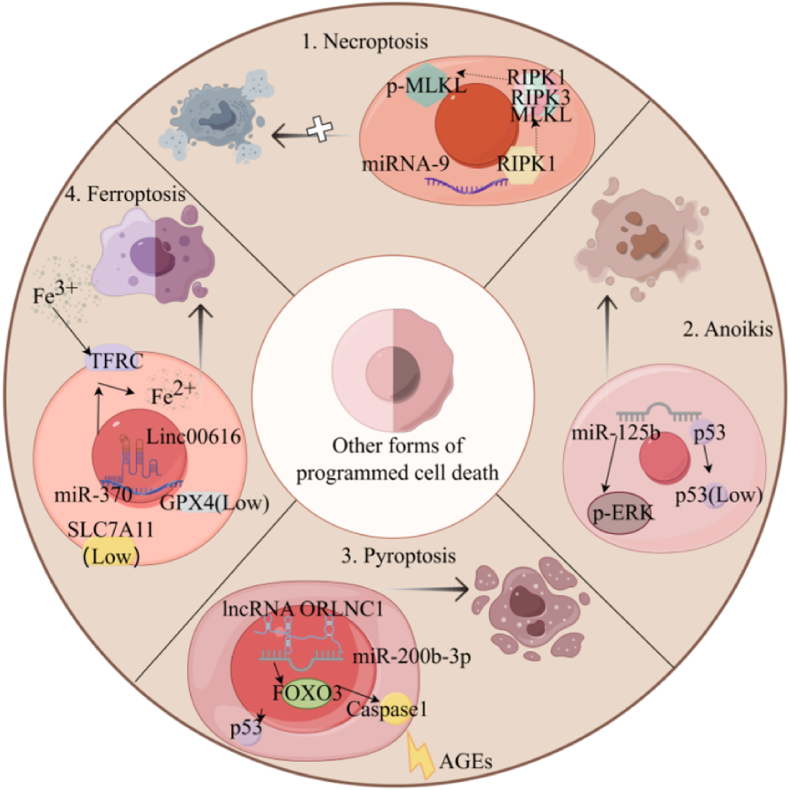
Some mechanisms of ncRNAs regulating other PCDs.

In addition to the well-known mechanisms of apoptosis and autophagy, ncRNAs are critically involved in the regulation of various forms of PCD, including stem cell necroptosis, anoikis, pyroptosis, and ferroptosis (Table 3) (Fig. 3).

(1) The miR-9 molecule specifically targets and downregulates the RIPK1 protein, leading to a diminished formation of the RIPK1-RIPK3-MLKL complex and a consequent reduction in phosphorylated MLKL protein levels. This regulatory mechanism inhibits the process of necroptosis;
(2) miR-125 targets the p53 protein, resulting in decreased expression of p53 and elevated levels of p-ERK protein under conditions of elevated miR-125 expression. This molecular mechanism promotes BMSCs resistance to anoikis induction during suspension;
(3) Under the influence of advanced glycation end products (AGEs), the expression of lncRNA ORLNC1 in BMSCs is upregulated, functioning as a ceRNA for miR-200b-3p and thereby indirectly modulating the activity of FOXO3. Concurrently, this upregulation of lncRNA ORLNC1 results in increased levels of p53 protein and Caspase 1 protein, ultimately inducing pyroptosis;
(4) The onset of periodontitis induces the up-regulation of linc00616 and the down-regulation of miR-370, which subsequently elevates Transferrin Receptor 1 (TFRC) protein levels. This molecular alteration enhances the cellular uptake of iron ions, thereby promoting ferroptosis in PDLSCs.

**Table 3:** The role and mechanism of ncRNAs in regulating other forms of PCD in stem cells.

ncRNAs	Role	Mechanism of action	Reference
miRNA-9	Inhibits necroptosis	Down-regulates of RIPK1, RIPK3, MLKL, and p-MLKL expression	[73]
miR-125b	Inhibits anoikis	Increases ERK phosphorylation and inhibits p53 expression	[74]
H19	Induces pyroptosis	Increases the mRNA and protein levels of NLRP3	[75]
lncRNA ORLNC1	Promotes pyroptosis	As a ceRNA of miR-200b-3p, it can indirectly regulate FOXO3	[76]
lncRNA SNHG1	Promotes pyroptosis		[77]
lncRNA IGF2-AS	Inhibits pyroptosis	Regulates dNTP concentration	[78]
miRNA-762	Inhibits pyroptosis	Down-regulates of IL-1β expression	[79]
linc00616	Promotes ferroptosis	Targets and inhibits of miR-370	[80]

#### 2.3.1 The role of ncRNAs regulate necroptosis of stem cells in inflammation

Necroptosis is mediated by an extrinsic signal that initiates a cascade involving the sequential activation of receptor-interacting protein 1 (RIP1), RIP3, and mixed lineage kinase domain-like (MLKL) signaling pathways [81]. ncRNAs possess the capability to modulate these receptor activity, thereby laying the groundwork for the development of novel therapeutic strategies in disease management [[82], [83], [84]].

Under inflammatory or other pathological conditions, stem cells are frequently accompanied by necroptosis [[85], [86], [87], [88]], and the inhibition of necroptosis plays a crucial role in tissue regeneration and recovery of organ function [89]. For example, a study by Song et al. demonstrated that the therapeutic efficacy of stem cell transplantation for acute pancreatitis was markedly improved by the expression of miRNA-9 in BMSCs. Subsequent investigations revealed that miRNA-9 directly targets RIPK1 [73]. In addition, HE J et al. conducted a comparative analysis between individuals with periodontitis and healthy controls, revealing 10 lncRNAs associated with core necrosis in human periodontal ligament cells (hPDLCs) which containing PDLSCs, including MALAT1 and EPB41L4A-AS1 [90]. Each of these identified lncRNAs has the potential to serve as a candidate regulator for periodontitis. These findings highlight the potential of targeting ncRNAs for therapeutic interventions against diseases characterized by necrotic apoptosis.

#### 2.3.2 Targeting ncRNA-mediated anoikis regulation enhancing stem cell transplantation efficacy

Anoikis constitutes a distinct form of PCD induced by the disruption of cellular interactions with the extracellular matrix and adjacent cells. This process is integral to embryonic development, the maintenance of tissue homeostasis, and the pathogenesis of various diseases. In the context of stem cell transplantation, insufficient matrix support and compromised adhesion to the extracellular matrix substantially contribute to the limited survival rates observed [91]. Concurrently, the study of anoikis presents potential for improving the effectiveness of stem cell therapy. And the adhesion to the extracellular matrix is crucial in guiding MSCs towards differentiation into specific lineage cells. Notably, the expression of miR-125b demonstrates an inverse correlation with cell-matrix adhesion levels and effectively targets the p53 protein. In a cellular suspension, miR-125b impedes anoikis by enhancing the phosphorylation of extracellular regulated ERK and suppressing p53 expression. However, upon re-adherent culture, miR-125b levels rapidly decline, thereby reinstating sensitivity to anoikis [74]. Therefore, the regulation of ncRNAs on anoikis can be utilized to enhance the *in vitro* adhesion of stem cells, thereby augmenting their cellular activity and proliferative capacity.

#### 2.3.3 The role of ncRNAs regulate pyroptosis of stem cells in disease development and stem cell therapy

Pyroptosis, also known as inflammatory necrosis of cells, is characterized by persistent cellular swelling that ultimately culminates in the disruption of the cell membrane and the subsequent release of intracellular contents, thereby eliciting a robust inflammatory response [92]. The NLRP3/Caspase-1/Gasdermin D (GSDMD) signaling pathway exemplifies a prototypical mechanism of pyroptosis and plays crucial regulatory roles in the initiation of pyroptotic cell death [93,94].

ncRNAs can modulate the aforementioned signaling pathways either directly or indirectly, thereby exerting regulatory control over pyroptosis. This regulation plays a crucial role in stem cell biology and therapeutic applications, providing novel foundations and strategies for managing related disorders [95,96]. For example, hyperbaric oxygen (HBO) therapy has been shown to positively influence stroke treatment by down-regulating the expression levels of H19. Research by Ye et al. revealed that H19 functions as a molecular sponge for miR-423-5p, thereby inhibiting its expression and subsequently targeting NLRP3. This interaction results in increased mRNA and protein levels of NLRP3, ultimately leading to pyroptosis of neural stem cells following hypoxia/ischemia [75]. Advanced glycation end products (AGEs) have been identified as inflammatory activators of NLRP3. In the presence of AGEs, the expression level of lncRNA ORLNC1 is upregulated. Acting as a competing endogenous RNA for miR-200b-3p, it indirectly modulates FOXO3. Elevated levels of miR-200b-3p suppress cell pyroptosis; however, simultaneous increased expression of ORLNC1 or FOXO3 enhances NLRP3 and Caspase1, thereby promoting pyroptosis in BMSCs [76]. lncRNA SNHG1 also has a similar effect [77]. In the investigation of sepsis pathogenesis, the study has demonstrated that the lncRNA IGF2-AS plays a pivotal role in regulating deoxyribonucleoside triphosphate (dNTP) metabolism, mediated by Homo sapiens high mobility group AT-hook 1 (HMGA1). Suppression of IGF2-AS expression results in the disruption of enzymes closely associated with dNTP metabolism, including decreased expression of Ribonucleotide reductase M2 (RRM2), Thymidine Kinase 1 (TK1), and Thymidylate Synthetase (TYMS). This perturbation consequently leads to elevated dNTP concentrations. These modifications promote the pyroptosis of EPCs in septic patients. Subsequent investigations have demonstrated a positive correlation between the expression of HMGA1 and its interaction with TYMS. These findings propose novel therapeutic strategies for the management of sepsis, including targeting IGF2-AS as a potential therapeutic intervention and considering the modulation of dNTP concentrations to regulate sepsis pathogenesis [78]. Moreover, the occurrence of acute mortality following stem cell transplantation markedly affects the therapeutic efficacy of stem cell interventions for infarcted myocardium. In a study by Chang Youn Lee et al., it was demonstrated that miRNA-762 can interact with IL-1β. The introduction of miRNA-762 into human adipose-derived stem cells (hASCs) significantly downregulates IL-1β expression under conditions of pyroptosis-induced stress. This modulation not only enhances the survival rate of transplanted hASCs but also mitigates cardiac fibrosis following ischemic injury, thereby improving overall cardiac function [79]. In summary, ncRNAs can facilitate the induction of pyroptosis in stem cells through the classical signaling pathway associated with pyroptosis, thereby contributing to the development of diseases. Simultaneously, we can harness the regulatory influence of ncRNAs on pyroptosis to augment the survival and therapeutic capacity of stem cells during stem cell transplantation.

#### 2.3.4 Ferroptosis

Ferroptosis is a form of programmed cell death driven by iron-dependent lipid peroxidation, setting it apart from other cell death mechanisms such as apoptosis, necrosis, and autophagy in terms of morphology, biochemistry, and genetics. It is characterized by the excessive accumulation of lipid peroxides and the destruction of the cell membrane system, resulting from the imbalance of intracellular metabolic pathways. These phenomena are intrinsically tied to abnormal iron metabolism and the disruption of lipid homeostasis. The Solute Carrier Family 7 Member 11(SLC7A11) and glutathione peroxidase 4 (GPX4) are function as crucial regulator of ferroptosis. Cellular uptake of cystine is mediated by the cystine-glutamate antiporter (System Xc-) composed of a light chain (SLC7A11) and a heavy chain (4F2hc). This imported cystine serves as an essential precursor for the biosynthesis of glutathione (GSH), a key intracellular reducing agent. GPX4 can use GSH as a substrate to reduce lipid peroxides to normal phospholipids [97,98]. These molecular components also play a crucial role in maintaining the lipid redox balance of stem cells and exerting inhibitory effects on ferroptosis [99], when ferroptosis occurs in stem cells, distinct alterations can be detected [100]. ncRNAs have been documented to influence the regulation of ferroptosis by modulating these critical molecular components [97,101,102]. In addition, iron transporters are essential for maintaining appropriate intracellular iron concentrations [103].

##### 2.3.4.1 The role of ncRNAs regulate ferroptosis of stem cells in inflammation

Ferroptosis involves various signaling pathways associated with disease [104]. For instance, The transplantation of MSCs activates the host nuclear factor erythroid 2-related factor 2 (Nrf2) signaling pathway, resulting in the inhibition of ferroptosis and a subsequent reduction in severe acute pancreatitis-associated acute lung injury (SAP-ALI) [105]; The up-regulation of Linc00616 in PDLSCs following periodontitis and Porphyromonas gingivalis lipopolysaccharide (LPS-PG) treatment has been observed. Additionally, LINC00616 not only downregulates the expression of GPX4 and SLC7A11 but also targets miR-370, indirectly influencing and enhancing the levels of TFRC, thereby promoting the occurrence of ferroptosis, LINC00616 knockdown may be a promising therapeutic strategy for periodontitis [80]. In summary, in the treatment of inflammation, the use of ncRNAs to regulate stem cell ferroptosis can better improve the therapeutic effect.

However, limited research has been conducted on the phenomenon of ferroptosis in stem cells. Further investigation is warranted to elucidate the underlying mechanism and provide clinical recommendations for the management of various diseases.

## 3 Summary and prospect

In summary, ncRNAs, particularly lncRNAs, miRNAs and circRNAs, play a crucial role in the pathogenesis and progression of PCD in stem cells. Numerous inhibitors have shown significant regulatory effects on the occurrence and progression of PCD in stem cells [36,106]. They primarily exert their regulatory functions by modulating the expression of ncRNAs, finely regulating proteins, and orchestrating dynamic changes in various pivotal signaling pathways (such as AKT, TGF-beta/smad, Wnt/beta-catenin, and mTOR), thereby promoting or inhibiting apoptosis, autophagy, and ferroptosis. However, current research on the regulation of stem cells by ncRNAs has predominantly concentrated on apoptosis and autophagy, with limited exploration of other forms of PCD. For example, while lncRNA H19 has been documented to inhibit stem cell apoptosis, autophagy, and pyroptosis, its involvement in anoikis and ferroptosis remains unexamined. Moreover, aside from the well-characterized ncRNAs such as lncRNAs, miRNAs, and circRNAs, the roles of other ncRNA species, including small nucleolar RNAs (snoRNAs), very long intergenic non-coding RNAs (vlincRNAs), and transfer RNAs (tRNAs), in stem cell PCD have received minimal to no scholarly attention. Zhang M et al. have identified a snoRNA-tRNA interaction network that influences ESCs differentiation; however, the mechanisms underlying its impact on ESCs apoptosis remain unclear [107]. In light of these deficiencies, we anticipate that future researchers will undertake comprehensive investigations to analyze the stories of ncRNAs and multiple PCDs in stem cells.

Some studies have challenged traditional paradigms by revealing that some ncRNAs containing short open reading frames (sORFs) in stem cells possess the potential to encode functional peptides or proteins [108]. These encoded small peptides or microproteins play a critical role in regulating stem cell biological activities. For example, Bernardo et al. reported the identification of 35 sORFs within 15 lncRNAs in human adipose-derived stem cells (hASCs), which potentially encode functional microproteins linked to the self-renewal capacity of hASCs [109]; Toddler, a small peptide encoded by lncRNA LOC100506013, is critical for embryonic development and is closely associated with gastrulation motility, as well as the migration of endodermal and mesodermal cells. And Toddler functions as a novel endogenous agonist of G protein-coupled receptors and plays a pivotal role in the regulation of cardiovascular system development and humoral homeostasis [110]. In addition, NEMEP encoded by lncRNA Gm11549 is a single transmembrane micropeptide located on the cell membrane, which can regulate glucose absorption during the differentiation of stem cells into mesoderm [111]; Neuronal differentiation of mESCs involves the partial redistribution of lncRNA Malat1 from the nucleus to cytoplasm, where it encodes the M1 peptide. Notably, M1 peptide activity increases proportionally with neuronal cell activity [112]. In parallel, the TUNAR microprotein-translated from TUNAR lncRNA and localized to the endoplasmic reticulum as a transmembrane protein-modulates neuronal differentiation by interacting with SERCA2 calcium transporter and regulating intracellular calcium dynamics [113]; Furthermore, LINC00961 is up-regulated during the differentiation of hESCs into endothelial cells. LINC00961 encodes a small peptide named SPAAR, which binds to the actin-binding protein SYNE1, while LINC00961 interacts with the g-actin sequestering protein thymosin β-4x (Tβ4). This mechanism is essential for the regulation of angiogenesis [114]. Given the close association between stem cell differentiation and PCD, these small peptides or microproteins may also regulate PCD during stem cell differentiation. And they can modulate proteins or signaling pathways closely linked to the regulation of stem cell PCD, making them potential key targets for stem cell therapy. For instance, in acute skeletal muscle injury, LINC00961 is down-regulated, and its encoded peptide SPAAR localizes to late endosomes/lysosomes, where it interacts with lysosomal v-ATPase and regulates mTORC1 activation through amino acid signaling. In the absence of SPAAR in the tibialis anterior muscle, mTORC1 is activated, enhancing stem cell proliferation and differentiation [115]. Since mTORC1 is known to regulate stem cell apoptosis [116] and autophagy [117], the SPAAR peptide encoded by LINC00961 likely plays a significant role in the regulation of stem cell PCD; In patients with steroid-induced osteonecrosis of the femoral head (SONFH), the expression of lncRNA DGCR5 is up-regulated. DGCR5 encodes a 102-amino acid small peptide, RIP, which promotes adipogenic differentiation of BMSCs, triggers osteocyte apoptosis, and accelerates SONFH progression. RIP binds to the N-terminal motif of RAC1, replacing lncRNA DGCR5 and inactivating the RAC1/PAK1 signaling cascade. This results in decreased phosphorylation of β-catenin at Ser675, reduced nuclear localization of β-catenin, and a shift in BMSC differentiation toward adipogenesis [118]. Importantly, β-catenin is also a critical regulator of stem cell apoptosis [[119], [120], [121]]. These studies indicate that ncRNAs regulate stem cells not only through their intrinsic functions but also by encoding small peptides or microproteins. Whether these proteins or micropeptides modulate PCD in stem cells, and the pathophysiological significance of such regulation in disease initiation, progression, and therapeutic intervention, requires further experimental validation and investigation.

The regulatory network of these ncRNAs regulating PCD not only influences differentiation potential, but also cell cycle dynamics, and senescence of *in vitro* cultured stem cells. And it contributes to the pathogenesis of various diseases. Consequently, ncRNAs are anticipated to serve as biomarkers to verify the effectiveness of disease diagnosis and clinical trials [[122], [123], [124]], as well as therapeutic targets and mechanisms of first-line drugs [125,126]. For instance, the assessment of ncRNA alterations has been proposed during the evaluation of the safety and efficacy of stem cell therapy [127,128]. This approach could serve as a potential marker for identifying and distinguishing patients' responses to clinical stem cell treatment [129], or as a biomarker for monitoring the therapeutic efficacy of stem cell interventions [130]. Furthermore, the identification of ncRNAs associated with PCD in both healthy individuals and those affected by diseases presents significant potential for early disease detection and the development of stem cell-based therapeutic strategies [90]. Consequently, research efforts can be directed towards the advancement of stem cell and RNA-binding therapies, which show promise as innovative approaches for future disease treatment.

To date, a diverse array of RNA-based therapies has received clinical approval, demonstrating efficacy in achieving therapeutic objectives through mechanisms such as gene silencing, gene up-regulation, and the regulation of cytokine expression. Compared to other molecular or protein-based drugs, RNA therapies exhibit a greater simplicity in design [131]. However, challenges such as precise targeting, efficient administration and heterogeneity continue to impede the clinical translation of RNA therapy and stem cell-based combination therapy. And when utilizing or applying ncRNAs for the regulation of stem cells, it is imperative to elucidate their precise functional mechanisms. For instance, in the treatments for obesity, Pengfei Gao et al. discovered that miR-128 exhibits a dual role by promoting autophagy while concurrently inhibiting apoptosis [132]. These observation suggests that apoptosis, autophagy, and other forms of PCD may constitute a coordinated cell death system, wherein one pathway can flexibly compensate for another. Consequently, further research is required to elucidate the precise role of this ncRNA. Additionally, as previously discussed, the integration of ncRNAs with protein biomarkers or metabolites should be considered to advance methodological approaches for the utilization of ncRNA as a biomarker in clinical applications.

## CRediT authorship contribution statement

**Ziling Liao:** Writing – original draft. **Weidong Liu:** Visualization. **Lei Wang:** Validation. **Wen Xie:** Data curation. **Chaoyan Yao:** Data curation. **Qianping Huang:** Data curation. **Xingjun Jiang:** Writing – review & editing. **Caiping Ren:** Writing – review & editing.

## Ethics approval and consent to participate

Not applicable.

## Availability of data and materials

Not applicable.

## Funding

This review was supported by the 10.13039/501100001809National Natural Science Foundation of China (82471424, 82071399, 30871246).

## Declaration of competing interest

The authors declare that they have no known competing financial interests or personal relationships that could have appeared to influence the work reported in this paper.

## Abbreviations

ncRNAs: non-coding RNAs; lncRNA: long-coding RNA; miRNA: microRNA; circRNA: circular RNA; PCD: programmed cell death; ceRNAs: endogenous competitive RNAs; BAX: CL-2 associated X; BAK: BCL-2 antagonist/killer; Cyt C: cytochrome C; Apaf-1: apoptotic protease activating factor 1; TNFR1/TNFα: tumor necrosis factor; IGF2BP1: insulin-like growth factor 2 mRNA binding protein 1; β-TrCPs: ubiquitin ligase-β-transducin repeat-containing protein; p-PKC: Phosphorylated Protein Kinase C; ERK: Extracellular Signal-Regulated Kinase; ESRG: embryonic stem cell related gene; hESCs: human embryonic stem cells; MCM2: minichromosome maintenance complex component 2; TRAFIP: TRAF Interacting Protein; DDX5: DEAD-box helicase 5; TDP43: TAR DNA-binding protein; NSPCs: neural stem/progenitor cells; KLF4: Krüppel-like factor 4; Sirt1: Sirtuin 1; Ndel1: neurodevelopmental protein 1-like 1; LEF1: Lymphoid enhancer-binding factor 1; NSCs: neural stem cells; YBX1: Y-box binding protein 1; HNRNPA1: heterogeneous ribonucleoprotein A1; mESCs: mouse embryonic stem cells; AP: alkaline phosphatase; BMP: Bone morphogenetic protein; AVEN: apoptosis and caspase activation inhibitor Gene; BNIP3L: BH3-only proapoptotic factor; PCNA: proliferating cell nuclear antigen; RASA1: RAS p21 protein activator 1; MAP3K1: mitogen-activated protein kinase kinase kinase 1; PBX1: PBX homeobox 1; PROK2: prokineticin 2; CDH1: cadherin 1; BIRC5: baculoviral IAP repeat containing 5; ROS: reactive oxygen species; STX17: Syntaxin 17; SESN2: Sestrin 2; TSC2: Tuberous Sclerosis Complex 2; PDCD4: programmed cell death protein 4; Mef2c: Myocyte Enhancer Factor 2C; PDLSCs: periodontal ligament stem cells; FAK: focal adhesion kinase; PDK1: 3-phosphoinositide-dependent protein kinase-1; P62: sequestosome 1; HDAC3: histone deacetylase 3; EPCs: endothelial progenitor cells; AMPK: AMP-activated protein kinase; MMP-1: matrix metallopeptidase 1; ATRA: all-trans retinoic acid; FGSCs: female germline stem cell lines; PTBP1: polypyrimidine tract-binding protein 1; OM-MSCs: olfactory mucosa-derived mesenchymal stem cells; WJ-MSCs: Wharton jelly multipotent stem cells; Syt1: Synaptotagmin 1; AGEs: advanced glycation end products; TFRC: Transferrin Receptor 1; RIP1: receptor-interacting protein 1; MLKL: mixed lineage kinase domain-like; GSDMD: Gasdermin D; HBO: hyperbaric oxygen; dNTP: deoxyribonucleoside triphosphate; RRM2: Ribonucleotide reductase M2; TYMS: Thymidylate Synthetase; hASCs: human adipose-derived stem cells; Nrf2: nuclear factor erythroid 2-related factor 2; SAP-ALI: severe acute pancreatitis-associated acute lung injury; GPX4: Glutathione peroxidase 4; CBL: Calcineurin B-Like Protein; mTOR: mammalian target of rapamycin; ATG autophagy related gene; SHED: stem cells from human exfoliated deciduous teeth; SCAPs: stem cells from the apical papilla; hPDLCs: human periodontal ligament cells; sORF: sshort open reading frames; SONFH: steroid-induced osteonecrosis of the femoral head; Tβ4: thymosin β-4x.
